# Facial Herpes Zoster With Hutchinson's Sign Complicated by Secondary Bacterial Superinfection: A Case Report

**DOI:** 10.7759/cureus.103969

**Published:** 2026-02-20

**Authors:** David Ramirez-Castro, Juan F Daza Ovalle, Daymara Boucle, Patricia Zorrilla, Juan Alonso

**Affiliations:** 1 Internal Medicine, Marqués de Valdecilla University Hospital, Santander, ESP; 2 Faculty of Medicine, Universidad de los Andes, Bogota, COL; 3 Neurology, Montefiore Medical Center, The Bronx, USA

**Keywords:** bacterial superinfection, diagnostic delay, herpes zoster ophthalmicus, herpes zoster virus, hutchinson's sign, ophthalmic complications, trigeminal nerve, valaciclovir

## Abstract

Herpes zoster ophthalmicus results from the reactivation of latent varicella zoster virus involving the ophthalmic branch of the trigeminal nerve and may lead to severe ocular complications. Hutchinson's sign, defined by vesicular or crusted lesions on the nasal tip, is a key predictor of ocular involvement.

We describe a 57-year-old woman with no relevant past medical history who presented with a 15-day history of pruritus and burning in the right eye. She was initially misdiagnosed with blepharitis and later periorbital cellulitis, receiving topical tobramycin and systemic antibiotics without improvement. On further evaluation, necrotic crusted lesions on the nasal tip and right frontal region were observed. The integration of clinical features, including dermatomal involvement, nasal tip lesions, and necrosis, established the diagnosis of herpes zoster with associated bacterial superinfection. Oral valaciclovir and clindamycin were initiated, resulting in a favorable clinical response with the progressive resolution of lesions and no ocular complications, underscoring the benefit of combined antiviral and timely antibiotic therapy.

This case highlights the importance of early recognition of Hutchinson's sign and timely initiation of antiviral therapy to prevent visual sequelae while also emphasizing the need to identify and adequately manage bacterial superinfection in herpes zoster lesions, including the prompt initiation of appropriate antibiotic therapy when indicated.

## Introduction

Herpes zoster results from the reactivation of latent varicella zoster virus and typically manifests with neuropathic pain and vesiculopapular eruptions in a dermatomal distribution [1]. The prodrome includes headache, photophobia, malaise, and dermatomal pain, followed by an erythematous rash that progresses to grouped vesicles with localized itching and severe pain [1,2]. Pain characteristics may vary and include paresthesia, dysesthesia, allodynia, or hyperesthesia [1].

Although thoracic involvement predominates, other regions may be affected, including cranial dermatomes, most notably the trigeminal nerve, as well as lumbar and cervical regions. Cutaneous lesions typically evolve over 3-5 days and progress to crusting within 7-10 days [1,3,4]. Herpes zoster ophthalmicus, involving the ophthalmic branch of the trigeminal nerve, may cause severe complications such as keratitis, uveitis, acute retinal necrosis, retinal scarring, and irreversible visual loss [5,6]. While female sex and smoking have been associated with earlier onset and increased risk, the most important risk factors for herpes zoster ophthalmicus remain advanced age and immunosuppression [5,7-10].

Hutchinson's sign is defined by vesicular or crusted lesions usually on the nasal tip, which reflects nasociliary nerve involvement and strongly predicts ocular complications [5]. Bacterial superinfection occurs when disrupted skin barriers from vesicles or ulcerations allow colonization by skin or environmental bacteria. Secondary bacterial superinfection of cutaneous lesions, most commonly due to *Staphylococcus aureus *or* Streptococcus pyogenes*, may mimic or coexist with periorbital cellulitis [11,12].

## Case presentation

A 57-year-old previously healthy woman presented with a 15-day history of pruritus and paresthesia affecting the right eye. At the initial evaluation, she was prescribed artificial tears.

**Figure 1:**
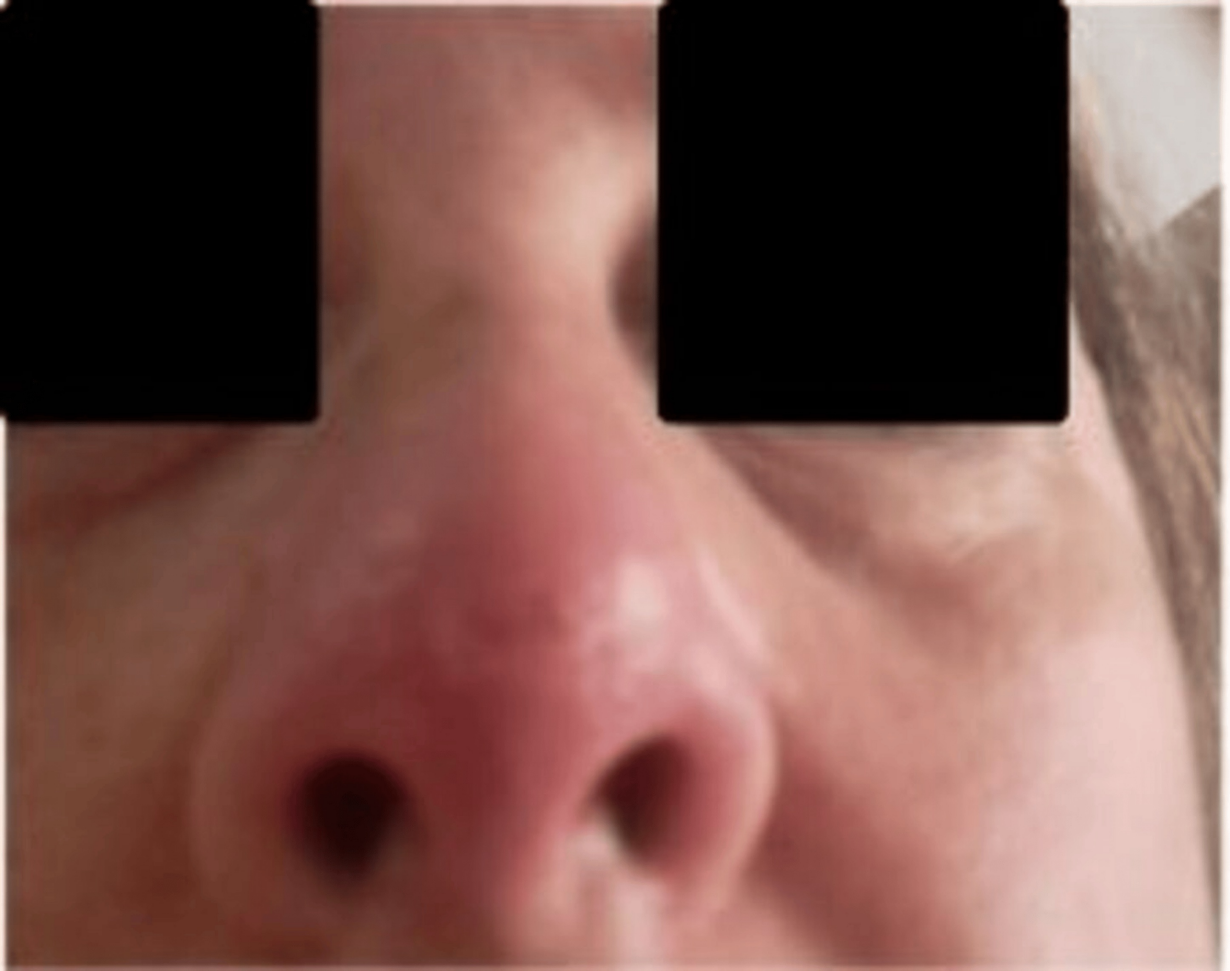
Initial phase of symptoms Initial presentation showing erythema and swelling involving the nasal tip prior to diagnosis. The patient consented to have her identity revealed, and a written and signed consent statement was provided to the journal.

Three days later, she developed nasal congestion and erythema involving the nasal tip and right eyelid. A diagnosis of blepharitis was made, and topical tobramycin was initiated. As advised, she reconsulted after 24 hours due to progression of erythema involving the nasal tip (Figure 1), forehead, and right eyelid, leading to reclassification as periorbital cellulitis and initiation of oral clindamycin.

**Figure 2:**
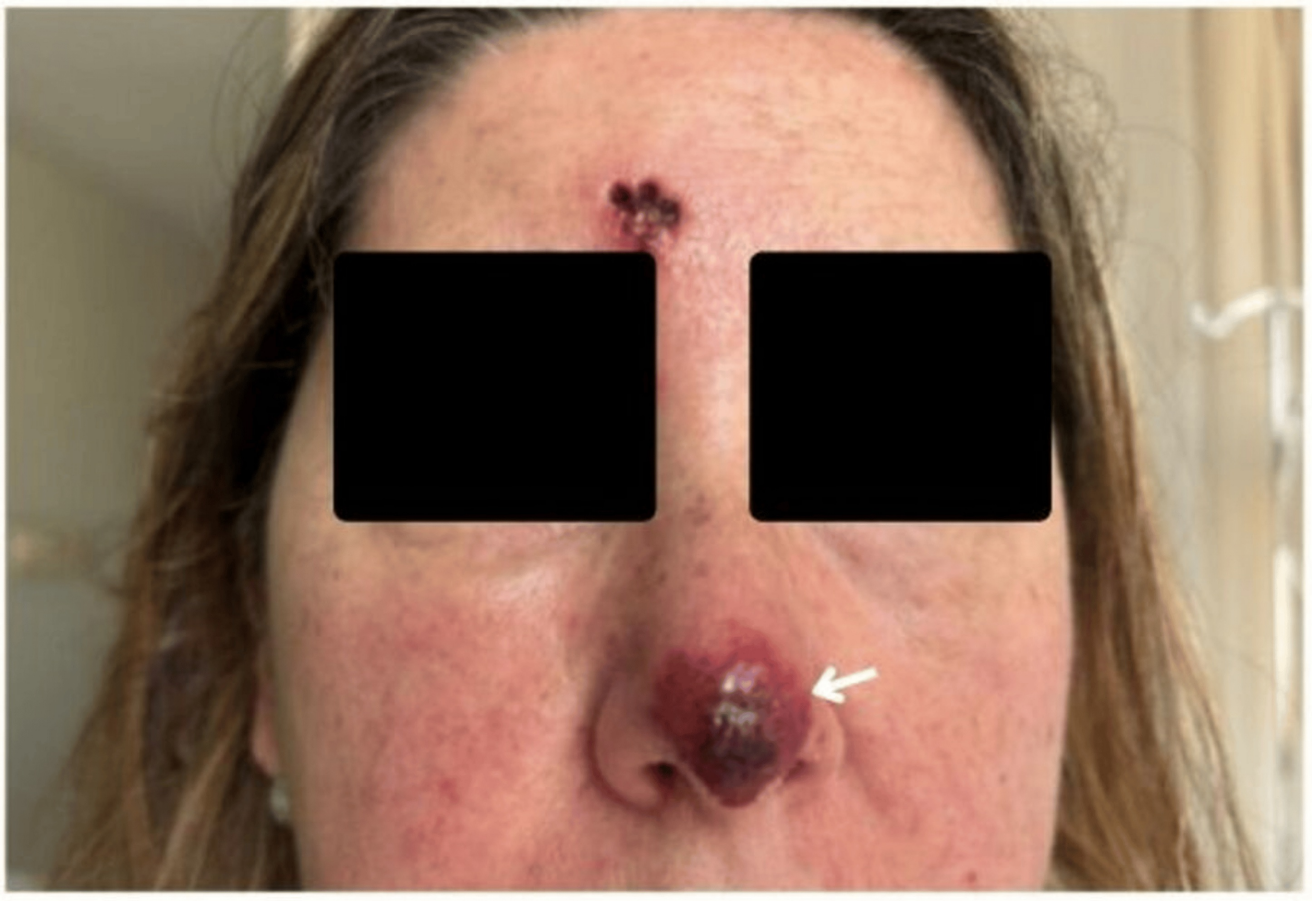
Hutchinson's sign Facial herpes zoster with Hutchinson's sign (white arrow) and concurrent soft tissue superinfection, demarcated by an erythematous halo of surrounding skin at the weekly follow-up visit. The patient consented to have her identity revealed, and a written and signed consent statement was provided to the journal.

At the weekly follow-up visit, necrotic crusted lesions (Figure 2) were identified on the nasal tip (~3 cm) and right frontal region (~2 cm). Ophthalmological examination excluded ocular complications. Based on the dermatomal distribution, the presence of Hutchinson's sign, and associated necrotic lesions, a diagnosis of facial herpes zoster with concurrent soft tissue superinfection was established.

**Figure 3:**
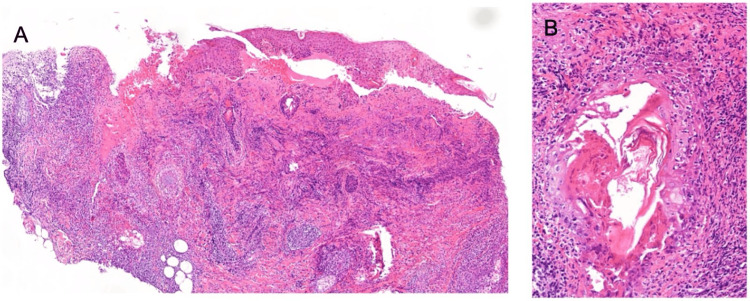
Histopathological examination of herpes zoster skin lesions (A) Skin biopsy demonstrating an intense, predominantly lymphocytic inflammatory infiltrate within the dermis, associated with marked folliculitis and focal epidermal erosion (H&E, ×4). (B) Higher-power view showing a more pronounced inflammatory infiltrate, highlighting dense lymphocytic infiltration (H&E, ×40). Image Credit: Courtesy of the Department of Pathology, Marqués de Valdecilla University Hospital, Santander, Spain

Oral valaciclovir was initiated due to its superior bioavailability and simplified dosing regimen compared to aciclovir. The diagnosis of varicella zoster virus infection was confirmed by polymerase chain reaction (PCR). Samples obtained from ulcerated and crusted lesions were collected for pathological evaluation (Figure 3A-3B). Microbiological confirmation of bacterial superinfection was not obtained, as antibiotic therapy had already been initiated; however, the diagnosis was supported by clinical findings, including necrosis and inflammatory changes, as well as the patient's favorable response to antibiotic therapy. 

**Figure 4:**
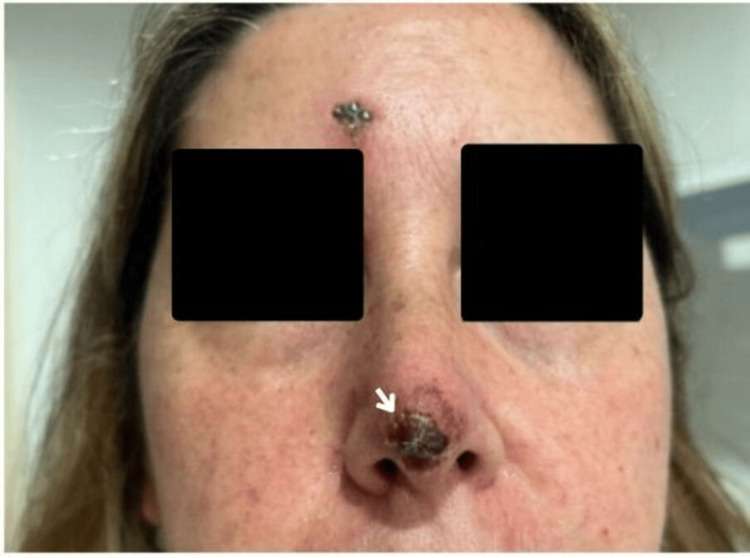
Phase of onset of symptom resolution at the final follow-up visit Clinical appearance during the early phase of symptom resolution, demonstrating reduction of erythema and progressive healing of cutaneous lesions with crust formation (white arrow) following the initiation of antiviral and antibacterial therapy. The patient consented to have her identity revealed, and a written and signed consent statement was provided to the journal.

At the final follow-up visit, the patient showed progressive clinical improvement, with resolution of erythema and healing of cutaneous lesions (Figure 4), and no ophthalmic complications were observed.

## Discussion

Recognition of Hutchinson's sign is a critical clinical marker, as it strongly correlates with nasociliary nerve involvement and increased ocular risk in herpes zoster ophthalmicus [13]. Early systemic antiviral therapy, ideally within 72 hours of rash onset, is essential to reduce complications.

This case is noteworthy for an atypical prodrome characterized by pruritus and paresthesia without early vesicles, which contributed to delayed diagnosis and initial mismanagement. Such presentations highlight the importance of considering herpes zoster in the differential diagnosis of unilateral periorbital erythema and neuropathic symptoms, even in the absence of classic vesicles.

Secondary bacterial infection of herpes zoster lesions is not universal but may occur in a subset of patients and is often under-recognized [12]. Clinical features such as necrosis, purulent exudate, or progressive erythema should raise suspicion and prompt initiation of systemic antibacterial therapy. In this patient, superinfection likely contributed to cutaneous necrosis, while timely antibiotic therapy facilitated recovery.

Valaciclovir offers practical advantages over aciclovir because of its superior bioavailability and simplified dosing, particularly in outpatient management [14]. The favorable response to combined antiviral and antibiotic therapy in this case emphasizes the importance of early antiviral treatment and vigilance for secondary bacterial superinfection, especially when necrosis or progressive erythema is present.

## Conclusions

This case illustrates the diagnostic challenges of atypical periorbital presentations of herpes zoster ophthalmicus. Hutchinson's sign is an important clinical marker that predicts nasociliary nerve involvement and increased risk of ocular complications. Diagnostic delay may occur when prodromal symptoms present without an initial vesicular eruption, making early recognition essential.

Necrosis or progressive erythema should raise suspicion for secondary bacterial superinfection, and prompt combined antiviral and antibiotic therapy is essential to reduce morbidity and preserve vision.
